# (Re)constructing identity following acquired brain injury: The complex journey of recovery after stroke

**DOI:** 10.1111/hex.13874

**Published:** 2023-09-20

**Authors:** Elena Faccio, Cristina Fonte, Nicola Smania, Jessica Neri

**Affiliations:** ^1^ Department of Philosophy, Sociology, Education and Applied Psychology University of Padua Padua Italy; ^2^ Department of Neurosciences, Biomedicine and Movement Sciences University of Verona Verona Italy

**Keywords:** acquired brain injuries, identity, narrative analysis, person‐centred care, positioning, public care, stroke

## Abstract

**Introduction:**

People with poststroke acquired brain injury (ABI) face a complex and often troubled identity reconstruction journey. The literature is rich with studies related to the psychological and neuropsychological components involved in rehabilitation, but it is lacking with respect to the investigation of the existential dimensions and the challenges associated with finding new senses and meanings for one's identity and future perspectives, body and interpersonal relationships.

**Methods:**

The aim of this study is to investigate the narrative processes of identity reconstruction after brain damage. Through a qualitative approach, 30 autobiographical narratives about self, body and the relationships with others were collected and analyzed. Semistructured interviews were used for the data collection. Narrative and positioning analysis were applied.

**Results:**

Four main positionings emerged: sanctioning a radical break with one's previous life; assuming a sense of salvation and compulsory as well as irreversible adaptation to the limitations associated with one's condition; feeling different and disabled; and considering new possibilities and active constructions of self‐being in relationship with others. These results underline the narrative processes of construction of the injury and the identity and delineate possible resources and instruments to improve the clinical practice for health practitioners. They are also valuable for other professionals who deal with neurological services and rehabilitation, such as psychological counselling and support for persons who have experienced ABI and their families.

**Patient or Public Contribution:**

This work resulted from a close collaboration between two universities and a hospital neurological rehabilitation department in the Veneto Region (Northern Italy). Three associations of people with stroke and their families living in the same area contributed to designing the research on the basis of the needs expressed by their members with the aim to identify strategies and devices to be implemented in the public service to improve the care pathway. They also participated in the interpretation of the data.

## 1 INTRODUCTION

Acquired brain injury (ABI) as cerebral damage occurring after birth and not a result of congenital or progressive disease, produces a wide variety of physical and psychological sequelae.^1^ ABI can result in different impairments that derive from either traumatic brain injury (e.g., physical trauma due to accidents, head injury, etc.) or nontraumatic injury due to internal or external sources (e.g., stroke, brain tumours, infection, hypoxia, ischaemia, encephalopathy, etc.).

This complex phenomenon has varied effects and impairments and involves different dimensions of the patient's life.^2^ Consequences often require an adaptation and reconstruction process around the person's situation, making that process a critical element in recovery, rehabilitation and return to social life. While the outcome of a certain injury depends largely upon the nature and severity of the injury itself, appropriate treatment and interpersonal resources play a relevant role in modulating the experience of the treatment and the return to everyday life.

Many studies in the literature highlight the impact of a brain injury on a person's life. Goldstein^3^, ^4^ underlined the strong emotional impact of ABI, calling it a ‘catastrophic reaction’ of functioning in the world. Caregivers, family and community‐based services emerge as central in postinjury rehabilitation, especially when physical issues become stable, medical care is no longer needed or available and identity shows its struggles.^5^

Among the possible brain injuries, the experience of stroke has been described in several studies as a rupture in everyday life, as a new aspect of life to which the individual must relate,^6^, ^7^, ^8^, ^9^ as an unexpected and overwhelming reversal^10^, ^11^; a critical severance^7^, ^12^ that produces a perception in stroke survivors of a foreign existence^6^; it also has a significant influence on personal temperament^12^ capabilities and abilities,^13^, ^14^ activities,^15^ roles and social relations^6^ and self‐identity.^16^, ^17^

Most studies on ABI have approached the topic from a medical or neuropsychological point of view. The complex psychosocial implications of an ABI are well documented, but how that injury impacts on identity and what this means to the adult remains relatively unexplored.^18^, ^19^ Despite this, some of the most enduring changes after ABI related to the subjective experience of the body, self and identity and to the relationships.^19^, ^20^, ^21^, ^22^, ^23^, ^24^

Relational approaches to identity have also emerged recently in neuropsychology and in the rehabilitation literature. Cloute et al.,^25^ through a discursive approach, have underlined the need to focus on how people with ABI are positioned and identified, for example, in a medical community (e.g., patient–expert, abnormal–normal, sick–healthy), understanding the implications of this on how a person understands himself. Other studies^26^, ^27^ highlight the impact of the labels attributed to them or assumed, such as the identification as ‘disabled’ or ‘survivor’ and the coping with these labels and roles. The process of managing the perceptions of others may be considered as crucial for identity.^28^, ^29^ Different positions or interpretative repertoires used by people after an ABI can inform the processes of construction and meaning‐making of the injury, their implications for identity, the rehabilitation process and interpersonal relationships. Nochi^26^ talked about the ‘loss of self’ by analyzing it in three areas: loss of clear knowledge of self (e.g., through memory loss), loss of self by comparison (e.g., of pre‐ and postinjury selves) and loss of self in the eyes of others.

Giving attention to narratives and self‐experience permits underlining and focusing on agency, that is, the possibility of active responses to a brain injury and not only the dependence on the characteristics of the biological damage. Furthermore, a narrative approach can help integrate the story of the individual's identity loss with the coconstruction of identity and thus project a new sense of self.^29^, ^30^, ^31^ The contribution of a narrative approach to identity is that it does not focus on a stable identity but on how people navigate this identity dilemma and the pragmatic implications, managing past and present into some more or less coherent whole, is echoed in narratives.^30^ This kind of attention can improve the analysis of the elements useful to integrate accounts into more satisfied life narratives, to project a new sense of self and to improve health in terms of biographical continuity, as proposed also by the life thread model.^32^

This knowledge can make a crucial contribution to both theory and practice psychology in dialogue with the field of neurological rehabilitation. This study seeks to explore the subjective perspectives through a qualitative approach and deepen the narrative processes of identity co‐ and reconstruction after brain damage.

### 1.1 Theoretical background

For the centrality attributed to self and identity as inextricably intertwined with language and narratives, the theoretical framework in this work is constituted by the perspectives of social constructionism^33^ and of symbolic interactionism.^34^, ^35^ They bring out a conception of identity as idiosyncratic and sociocultural construction, constantly negotiated in different contexts and interactions between individuals and in continuous change.^33^ Purely, in accordance with the above, the analysis of the ‘identity positioning’ looked the most suitable for achieving the topic of the research (see Table 3). Cerebral events, even in their origins and potential organic consequences, are considered shaped by the discourses and narratives that can be made of them both at the level of common sense and of the scientific community, as well as by virtue of specific idiosyncratic biographies and movements of identity.^36^, ^37^

### 1.2 Study aims

This research tries to answer questions such as: What is the impact of an ABI on the identity, particularly with stroke? How does it change the narration of the self, the body and the relationships with the others? What are the pragmatic implications in everyday life and when they are talking about their past and future?

Thus, we paid attention to the implications related to the descriptions of what they wanted to tell about their experiences both in terms of personal suffering and loss (of bodily functions, life perspectives and life experiences) and in terms of the possibility of openness to the new. indeed, we know that roles and relationships can be developed and fostered by taking into account the biological impact of ABI.

The research provides insight into the implications derived from the injury on identity and relationships to identify possible resources and tools to improve the clinical practice of psychologists but also other professionals involved in neurological and rehabilitation services.

## 2 MATERIALS AND METHODS

### 2.1 Contexts and participants

Participants were recruited through neurorehabilitation services in public or private hospitals and through associations dedicated to people affected by ABI in the Veneto region (in the north of Italy), in the city of Padua.

The goals of the research were co‐constructed and shared with the staff of the institutions involved in the research: the University of Padua (Department of Philosophy, Sociology, Pedagogy and Applied Psychology), the University of Verona (Department of Neuroscience, Medicine and Movement) and the Neurological Rehabilitation department of the public hospital of Borgo Trento in Verona. The inclusion criteria were: a diagnosis of a moderate or severe ABI after stroke; 18‐year‐old or above; at least 1 year after the injury; intention and capacity to sustain and interact verbally or in writing with the interviewer and the principal caregiver. In the eventuality of an aphasic patient, the caregiver has been involved to stimulate the patient. Knowledge of already consolidated communication strategies was fundamental for the management of interaction with the researcher. The selection criteria for the caregiver included the recognition of his/her role by the person with ABI, being husband/wife/partner, son/daughter or any other important or specified caregiver.

We decided to keep the range of the age wide, including people with different social and personal situations, different perspectives and goals, for instance returning to school, work or parenting experience.^38^, ^39^ We tailored interviews to help the patients to express their own needs.

The data collection was based on the principle of theoretical saturation, considering on one hand, the point where variation ceases, usually when the number of interviews reaches around 15 (±10)^40^ and on the other, evaluating the text collected to be meticulously analyzed.^41^ So, the number of participants was not predetermined, to collect and leave room for the variability of texts, biographies, and caregivers' relations. The final large numbers promised to obtain an exhaustive variability of texts and a detailed analysis of the discursive processes implied.

Three Veneto associations of people with stroke and their family (members of the A.L.I.Ce. association for the cities of Venice, Rovigo and Verona) collaborated by sharing the general aim: the improvement of the public care path quality. Potentially interested people were contacted by the neurorehabilitation department of Verona Hospital or by local associations. A total of 30 people were recruited with ischaemic and/or haemorrhagic stroke, five people refused to participate. Afterwards, participants were given the contact information of one of the researchers, so that they could share the details of the research, answer questions or any concerns and choose the most appropriate interview location.

Informed consent to the research, approved by the Ethics Committee of the School of Psychology of the University of Padua (3793), was shared with each of the participants.

Below is a summary in Table 1 of the demographic data of participants.

**Table 1:** Summary of demographic features of study participants.

Demographic data	Demographic features	Participants
Gender	Men	18
	Women	12
	Other	0
Age	18–40	3
	41–60	19
	61>	8
	Mean age: 54	
	Standard deviation: 15	
Origin	Verona	12
	Venezia	7
	Rovigo	10
	Other	1
Occupation	Employees	8
	Disabled/invalid	14
	Retired	5
	Student	2
	Other	1
ABI (stroke typology)	Ischaemic stroke	22
	Haemorrhagic stroke	8
Distance from the event	1 year	3
	1–2 years	6
	2–5 years	11
	>5 years	10

### 2.2 Data collection

Interviews were conducted from September 2020 to June 2021. Qualitative methods were privileged to highlight the personal, interpersonal and social theories used by people to give sense to the health issue derived by the particular brain damage.^42^, ^43^, ^44^ Data collection and analysis were conducted in accordance with the guidelines for rigour in qualitative research.^37^, ^41^

We preferred a particular form of semistructured narrative interview^45^, ^46^ since it allows attention to meanings and themes related to the dimensions of interest and at the same time leaves room for other remarkable aspects of the participants that might emerge in the coconstructed dialogue. The interview is not considered just as an instrument, but a method, because it involves an interactive process between interlocutors.^45^ Interviews were carried out at the headquarters of the associations involved (Rovigo) and remotely via telematic platforms or the telephone. The interviews have been audio (N.15) or video recorded (N.15) with the exception of one because of requests from participants, and transcribed entirely. Informed consent was made available to participants before the interview, it has been read and discussed together, answering doubts or questions. In the case of aphasic people, the caregivers facilitated the understanding of the step composing the process.

The interviews were conducted in person, at the association's headquarters (N.12 in Rovigo), and online (N.2 via phone calls and N.16 via Zoom or Google Meet). This flexibility in data collection made it possible to involve even those who would have declined to participate in person due to physical or logistical limitations. Both modalities chosen had their respective advantages and disadvantages. The face‐to‐face context may have made it easier for interviewees share dynamics and aspects related to the use of the body and space, an important theme for the topics under research. Similarly, there were those who preferred the online context, which for them may have facilitated the sharing of other aspects, such as those related to the use of the everyday space in one's private home, which were equally important with respect to the reflections expressed during the interview.

The interview covered the following:

1. The area of the self: The focus was on the narrative processes used to describe past, present and future perspectives, expectations and the pragmatic implications of these narratives.
2. The area of the barriers and opportunities considered and used to manage the injury: The focus was on the narrative processes used to describe them; their relationships with other and the daily‐life contexts; the process of past and new roles assumptions and the perspectives, expectations and critical issues in the relationship and in the professionals involved in the rehabilitation process.

Table 2 shows the main interview questions for each of the research areas.

**Table 2:** Interview questions.

Area of self	Positionings with respect to stroke.	How would you describe the experience of having a stroke?
		What impact did the stroke have on your life?
	Temporal positionings with respect to the injury (past, present).	How would you describe yourself before this event? How would you describe yourself today?
	Time positionings with respect to the injury (future).	How do you imagine yourself in the future?
Area of barriers and opportunities	Positionings with respect to managing the impact of injuries.	How have you handled this impact over time and to date?
	Positionings with respect to managing the critical implications of the injury.	What critical aspects have you identified in managing this experience?
	Positionings with respect to managing the strengths of the injury.	What strengths have you found in managing this experience?
	Positionings with respect to caregivers and other professionals involved in the path of care.	How would you describe your relationship with those closest to you in managing this experience (e.g., partners or relatives)?
		How would you describe your relationship with the health care professional(s) you work with in managing this experience?
	Positionings with respect to injury management from the perspective of professionals.	What suggestions would you give to psychologists or to rehabilitators for helping people who have experienced strokes?

### 2.3 Data analysis

In this study, identity is understood as an interactive, situational and relational process not separated from the cultural frame in which people are inserted. Identity does not mean self‐contained, and the focus is on the process of construction.^32^, ^36^ Narratives and discourses are retained as particularly important in meaning‐making and constructing and reconstructing identity.

A method of narrative analysis has been used together with the concept of positioning (see Table 3).^43^ Positioning theory refers broadly to the close examination of how speakers describe people and their actions in one way rather than another and, by doing so, attribute parts or fluid roles to the self and to other people in the discursive construction of personal stories and biographies.^43^, ^47^, ^48^

**Table 3:** Positioning theory and its application in research.

Theoretical assumptions of positioning theory	Positioning theory opens up a new dimension in the psychology of interpersonal encounters by drawing explicit attention to the role of rights and duties in the management of action. It is concerned with revealing the explicit and implicit patterns of reasoning that are realized in the ways that people act towards others. The fundamental insight on which positioning theory is based is the principle that psychology must primarily be the study of meanings.
Research questions it is aimed at answering	‘Why did they do that?’ ‘Why did he think that?’ ‘Why did she/he feel so and so?’ These questions are all qualified by the phrase ‘in the circumstances’. Positioning is the process of attributing parts or fluid roles to speakers in the discursive construction of personal stories. We focused on analyzing the identity movements that the participants adopted in their narratives in relation to their strokes. We also considered the wider sociocultural setting, which provides the backdrop for certain stances and ways of describing oneself, the practitioner and their clinical and family relationships.
Applications to the present research	All narratives were considered not as intrinsic characteristics but as situated and shared expressions that construct a ‘reality’ through dialogue and thus through the interaction between the participants and the researcher. The steps involved in positioning analysis are as follows: 1. Exploring every interview in its complexity, seeking to identify all the peculiar themes and narratives concerning the self in relationship with the stroke. 2. Tracing the themes and narrative elements common to all the narratives concerning the self. 3. Identifying the portions of texts relating to the interview enquiry areas. 4. Creating several categories of analysis in which the narrative modalities used for the description of the self can either be similar or extremely different. Each category is understood not only as a label that represents content emerging from the interviews, but also as a linguistic configuration of reality, and therefore, as such, processual, fluid and changing in space and time of speech and dialogue. 5. Each recognized position was re‐examined and, if necessary, recoded in new or existing positions. The narrative evolutionary lines between the groups of positionings were questioned and compared between the authors. The data could be continuously reinterpreted, and while we monitored the consistency within these narrative and discursive expressions, we also sought to remain faithful to the interviewees' stories and to their specific interactions.

In the case of participants with aphasia, the data collection process was significantly different. The caregiver facilitated the translation and interpretation of gestures and other nonverbal and bodily elements manifested during interactions with the interviewer.

As to the roles and backgrounds of the researchers, two of them worked closely with the caregivers considered in hospital wards, while the other two were concerned with the study of narrative and interactive identity construction processes, focusing on the impact of organic illnesses at the psychological level. The findings were shared with the associations that joined the project during three closing meetings. On these occasions, not only were the main results presented, but feedback and opinions were also sought with respect to the most significant findings. This made it possible to select and present in the article only those positionings that the members of the association considered most representative; other positionings (see Table 4), which are more marginal, are summarized in Table 4.

**Table 4:** Summary of main findings.

Areas of enquiry	The continuum along which positionings may be placed	Positionings
The self before and after a stroke.	From the identity split between before and after stroke to the discovery of priorities in life choices.	‘I am another person, and this is another life’.
		‘I can't accept myself’.
		‘I'm saved; I see life differently’.
The self in the future.	From passive anticipation to intentional activation.	‘I don't think about the future’.
		‘I hope to be useful and independent’.
		‘We must continue’.
Barriers encountered because of the injury.	From self‐assigned to heteroassigned judgement.	‘I see it in the eyes of others, and I feel different’.
		‘I lost people I did not expected to lose’.
		‘I have to ask for help—one gets lonely’.
Opportunities in managing injury relapses.	From self‐referentiality to coconstruction with others.	‘I’.
		‘My relationship with others’.
		‘Inventing new strategies’.

## 3 RESULTS

The participants' most common positionings towards the ABI (ischaemic or haemorrhagic stroke) are discussed below with word‐for‐word excerpts. These positionings were translated from Italian into English at the end of the research process, with an attempt to remain faithful to the original words and meaning.

The results are summarized in Table 4.

### 3.1 The self before and after stroke

Depending on the severity of the injury or the hemisphere involved, participants described themselves in the past as more capable and more active, with many interests and things to do. Some daily activities are no longer independently manageable, and the motor functions needed to perform them often cannot be rehabilitated. Paralysis, muscle weakness, aphasia and cognitive deficits are among the devastating results of strokes.

‘I can't accept myself’

Through this positioning, the lesion is given decisive power with respect to the possibilities and abilities that can be put in place to continue in one's biography. People described themselves as ‘disappointed’, as ‘ordinary people’, or as ‘people who no longer want anything’ and ‘who will never heal’. One's story around their brain injury establishes the impossibility of accepting their condition at this precise moment. The story is moved and centred on an exclusively sanitary dimension linked to the physical and organic consequences of the injury. These narrative modes are fixed and peremptory; nothing can change, and this is the necessary consequence of the event that has determined the future. At the same time, self‐acceptance is closely linked to the organic body. The discourses revolve around a bodily dimension in its health sense; they relate to as many discourses conveyed and present at the sociocultural level on the ability and criticality of failing to appear healthy or at a level judged normal.

‘I'm saved; I see life differently’

This positioning highlights two different facets: on the one hand, the significant impact of the injury in the story of the self as a person who has survived from its potentially deadly consequences, once again to emphasize the determining capacity of the same on biological and biographical life. On the other hand, it highlights the presence of modalities that contemplate other possibilities besides those of forcefully affirming the rupture from an earlier moment and the impossibility of accepting oneself. For example, difficulties and problems concerning various forms of so‐called disability can be understood, legitimized and streamlined by judgements linked to the performance of normality. Other people said that they have changed the way they see priorities, the rhythms of life, and their relationships with others. The event, however impactful, unforeseen and drastic in the change that accompanies it, is used as an opportunity to re‐evaluate priorities and values and, above all, to rebuild roles and relationships within the family or in other surrounding places. For the area of the self before and after stroke, it is possible to observe different positionings in a continuum moving from the identity split between before and after stroke to the reprioritisation of life choices.

### 3.2 The self in the future

The main positionings referring to the self in the future reveal the prediction of inauspicious and miserable scenarios—denying even the possibility of imagining it—because of the expected sequelae of the stroke, up to the inclusion of one's own involvement in imagining and planning one's future.

‘I don't think about the future’

Some participants failed to contemplate the idea of a future perspective. Although present as a possibility, it fails in being a specific imagination, a specific desire, because they are smoothed out by the narrated impact of the consequences of the injury in the present moment.

People defined themselves as ‘lost’, ‘bewildered’ or ‘fearful of the thought of the future’ for fear that such an event might happen again with lethal or worse outcomes, or that the sequelae of the injury may lead to worsening rather than even slight improvements.

‘I hope to be useful and independent’

Although most people with ABI are told in drastic terms of the changes and difficulties, they will have in carrying out activities or having satisfactory relationships, the issues of independence and usefulness are particularly relevant. Narratives refer to the supposed need to ‘be like others’ in terms of performance and appearing normal and self‐sufficient. On the other hand, there also emerges a way of being able to place oneself in the relationship with the other, within traditional roles and according to certain values or standards (e.g., preparing lunch, accompanying children to school, keeping accounts, being the main responsible party) but also being able to invent and build new roles. In this way, the biological limits, and the perception of being a victim of the event pass into the background, albeit remaining present as discourses, but leaving room for other goals, roles and relationships.

‘We must continue’

Such positioning highlights more than others the drive to do, change and perform using possible limits given by the injury and individual and interpersonal resources traced in one's own history. There may be a reference to the total or partial recovery of certain capacities, but above all, the reference to the intentionality of going ahead on one's own path, defining goals and life plans. The injury is not necessarily there in this story, where instead there is the contemplation of the possibility, the project and the development of agency both in the use of one's own body (e.g., doing physiotherapy; using physical activity to model the other goals of life) both in developing and maintaining relationships with others.

Briefly summarizing, for the area of the self in the future, it is possible to observe different positionings in a continuum moving from passive anticipation to intentional activation.

Barriers encountered because of the injury

What first characterizes and defines this area has to do with its content, that is, its relationships with others. These are considered fundamental. These positions move from a polarity that implies an external judgement introjected and made one's own—with discourses relating to normality and disability with impact on the identity—to another that distances the judgement from oneself and attributes it to the other, thus perceiving and sanctioning a difference between normal and disabled people.

‘I see it (my disability) in the eyes of others, and I feel different’

Through this positioning, the one who is brought into play is the other, a known other who was once close but has slowly moved away or an unknown other who simply crosses the street. In both cases, the gaze that is experienced and that of judgement of one's perceived disability: for the use of a stick to support oneself or a wheelchair to walk, for a stutter, for a difficulty in remembering programmes. It is told in terms of ‘embarrassment’, ‘compassion’, ‘defeat’, ‘diversity’ towards those who are put and legitimized in the position of being able to judge what is normal or not. The judgement narrated and experienced, whether it is effective, is introjected and done with the implication of affirming and validating a sense of diversity and division between itself and the other able‐bodied.

‘I lost people I did not expected to lose’

There are also criticalities related to the most intimate and close relationships. The ‘surprise’, ‘disbelief’, and ‘lack of expectation’ linked to the possible loss of some people is often told, along with the subsequent ‘isolation’ or the ‘difficulty of asking for help in case of need’. The role of the parent, the friend or the companion is considered relevant and at the same time loaded with expectations that in most cases have not been able to be fulfilled. Conversely, people who remain are regarded as great resources, for concrete aid, but also as a fulcrum to continue the road of rehabilitation rather than in the interpersonal roles to be played.

‘I have to ask for help, and one gets lonely’

This positioning refers to specific spaces, services, people and figures in health or social care that the protagonists may need. Regarding the use of public services, the interviews show how the pandemic restricted access to facilities, and this was experienced by people as particularly critical, as it created a difference between ‘first‐class patients’ (e.g., covid patients) who could use public services and others who could not. In addition, the lack of information about one's injury and its consequences is seen as severely limiting, even with respect to applying for other private facilities at which one can continue rehabilitation treatment after the public service. In this sense, people ‘feel alone and abandoned’, ‘left to their own devices’, ‘neglected’ with the risk that continuity of care will not be given.

For the area of barriers encountered, different positionings emerged that may be represented in a simplified way through a continuum from self‐assigned to heteroassigned judgement.

*Strengths in managing injury relapses*.

Positionings diversify according to various shades of agency and intentionality in the direction of a goal of building and managing struggles. Simultaneously, they move from a polarity that implies a reference to oneself as independent and able to face difficulties to a polarity that contemplates collaboration, coconstruction and the invention of new roles and strategies in dialogue with the other.

‘I’

A relevant positioning is that which sees the story revolve around one's own person as the main resource and reference point in the direction of performative scope. Expressions such as ‘having to fight’, or ‘go ahead or you are dead’ are representative of this positioning. At the same time, other facets of adaptation, effort and patience, tolerance, and references to managing a difficult situation emerge.

‘The relationship with the others’

Where present, the other is configured as an important resource. Whether it is the parent present every day by the side of the bed or the companion to help with daily hygiene, these are described as people who have strived, struggled, helped and strongly supported. Health workers are described in most cases as stern but as extremely useful in understanding the situation and the physical consequences of stroke. Especially in the immediate aftermath of a stroke, assistance is considered crucial and indispensable in being able to carry out daily activities, such as walking, preparing lunch, eating, dressing, washing. Later, when difficulties begin to be perceived as chronic, constant and patient support, but also stimulation, are crucial to continue to manage critical issues and create adaptations. Rehabilitation professionals and neurologists in particular are considered a real point of reference for clarifying health conditions and calibrating future expectations on the basis of the diagnosis and assessment of the impact of the stroke event. From their clinical assessment is derived the possibility of salvation or disillusionment.

‘Inventing new strategies’

The body is used even in its perceived limits, inventing new ways to carry out daily activities, jobs or tasks to be completed (e.g., strategies to be able to make a plate of pasta; remember appointments; walk on the street without being hindered; or put on a bra independently). Health figures seem to have a fundamental role in sharing and learning new strategies, but also in inventing specific strategies and shaping them. The implication, in addition to exploiting the agency's potential, which is inextricably linked to the narration and use of the body, is to foster a sense of satisfaction, esteem, responsibility and action in one's life.

For the area of opportunities in managing injury relapses, it is possible to observe different positionings on a continuum moving from self‐referentiality to coconstruction with others.

The positionings noted and explored so far seem to be very common among the participants, as emerged during the three discussion meetings with association members. This, despite the variability in demographic data. More specific and less representative positionings have been summarized in Table 5 since there is no space here to elaborate and discuss them in depth.

**Table 5:** Other emerging positionings.

Areas of enquiry	Other peculiar positionings	Examples
The self before and after stroke.	Other positionings are concerned with the possibility of giving continuity to one's biography without the interruptions caused by the stroke. These positionings highlight some awareness of the implications of the injury and their management.	‘I am the same as before, but with the hand brake pulled’.
The self in the future.	Other positionings involve predicting a future in which things can only get worse, going on to extend limitations to various areas of life beyond the physical implications of the injury, for example, in relational and social spheres.	‘I see a bleak future’.
Barriers encountered because of the injury.	In reference to other people with stroke, there emerges, on the one hand, a sense of belonging to the group, and on the other, a sense of humiliation and reflexive self‐criticism confirming a type of disability that, to a naive eye might not be visible or is only partially known (with an underestimation of the related implications and difficulties).	‘It's like we have to justify our disability’.
Opportunities in managing injury relapses.	Less frequently occurring positioning that is still generally considered relevant. This positioning is linked to a sense of self‐efficacy in managing minimal daily activities, even over a short time after an injury. It is used as a driving force for continuing to deploy strategies for adaptation and change.	‘Seeing that I was able to do things’.

Considering the results as a whole and relating them to the biographical variables considered, it is possible to say that the collected texts did not reveal any differences between men and women in terms of stroke‐related experiences. In contrast, gender very much comes into play with respect to roles such as caregivers, wives and mothers. Young people seem more inclined to be open to new ways of looking at the problem, while mature adults to closure or passive and resigned adherence to the effects of stroke. The absence of a stable job seems to be an element of vulnerability such that the person feels even more dependent and disabled. The more time has passed since the event, the greater the degree of acceptance and the construction of coping strategies as well as, in some cases, the narrative of limited possibilities for action.

## 4 DISCUSSION

Consistent with the literature,^6^, ^7^, ^8^, ^25^, ^27^ this study has found that the identity of people affected by stroke is built on narratives of drastic change, sense of loss of self and of one's own abilities and how these aspects bring with them pragmatic implications in terms of discomfort and ineffectiveness in different life contexts.

In the different areas of investigation, positions and narratives emerged that attribute to the event the cause of current problems that are seen as insurmountable and that are used to generate in the story an identity fracture between a time past and a time present. Connected to this type of dominant causal narrative is also a peculiar vision of the future, which in some cases is avoided and/or imagined as even more critical than the present. Unlike the other available studies, which focused on the rupture between past and present, our research also shifted its gaze to the relationship between present and future. Collected personal positions can be placed along a series of continuums ranging from not being able to imagine or being afraid of the future to the position of telling themselves to continue and/or having to get back to feeling useful for others.

Two basic elements emerged: on the one hand, the need for acceptance of one's condition, on the other hand, the need to ‘invent new roles’ to relocate oneself in the network of relationships. Indeed, to make the stories problematic are several common aspects: the sense of ineffectiveness in the management of daily life and the perception of uselessness and dependence on intimate people. Two other continuums of positionings may be identified from very limited consideration of the possibility of improvement to full confidence in it, and from the use of discourses about normal–abnormal binomial to the disability as a condition to be judged and which is judged, inferiorized and assisted by society.^26^, ^27^ Judgement and typing (disability; diagnosis) emerged as the most critical aspects, while closeness and patience in the face of a condition that is painful and difficult to accept were the most useful ones. This element is of paramount importance in the rehabilitation journey. The person may not be able to actively engage in the search for strategies to recover motor function until he or she stops fighting against what has happened and stops judging himself or herself. It is necessary to get back to feeling ‘normal’ (thus accepting), and to this end, confrontation, perhaps mediated or facilitated by health professionals, with ‘others like me’ may be of great importance. Although to a lesser extent, there were also narratives that contemplated the continuity of the biography: stroke as one of the many possible events of life.^25^ Further positionings and narratives emerged including a vision of possibilities towards the construction of other roles or the management of the critical fallout of the injury. To facilitate these processes are the narratives in which the story of the ‘identity fracture’ is not preponderant, but the agency of the person also emerges in the interaction with others, for example, in the construction of strategies for the management of daily life or in the repercussions of the injury on the body (hemiplegic, mnemonic or verbal). These strategies reveal the constructive character of their actions toward the future and the relevance of self‐description in terms of continuity and favourable development. Moreover, the positionings regarding relations with different people have emerged as particularly relevant: caregivers, health professionals and the generalized other. They highlight the relevance of engagement in interaction and in the definition of new roles and strategies that include the biological body and make it a relational body.^21^, ^49^, ^50^, ^51^

These findings show the potential of narrative analysis and positioning with respect to the construction of identity and the relationship between psychology and neurology. Moreover, they can help differentiate narratives that can facilitate rehabilitation and/or the construction of new roles and promote the overall health from narratives that lock and immobilize the person in his or her impairment.

The prevailing narratives touch on the judgement towards oneself, the comparison with the past, the contrast between a condition of pathology and disability and a condition of lost ideal and sought‐after normality. The narrative fracture given by the event is often presented as profound, with the risk of being considered unchangeable. It is precisely here that professionals can intervene by promoting the therapeutic alliance with patients, so as to foster the pursuit of targeted and shared therapeutic goals. Quality care relationships allow the practitioners to anticipate possible criticalities and to intervene on them to promote a strategic vision involving not only the body, but also the relational network available to the person.

## 5 CONCLUSION

The results show various outcomes. From a theoretical point of view, it is possible to highlight a conceptualization of identity as a linguistic and relational process^35^, ^52^, ^53^ inextricably connected to the dimension of the organic and natural body. This process is involved during the entire biography and can include not only the construction of the dimensions of emotions, thinking or interpersonal behaviour but also the meaning‐making of biological and neurological aspects related to one's own health condition. Furthermore, the results suggest the need for improvement of narrative approaches and methods in the field of brain injury. It could be useful to include narratives and subjective experiences of people with experience with ABI, who have traditionally been considered incapable of providing ‘accurate’ descriptions or sufficient dialogues,^54^ for inclusion in qualitative and psychological research.

The methods of analysis and the tools used highlight the plural and dynamic character of the identity, underlying both the main critical and the useful aspects of the biography. We believe that narratives may also have great potential in the process of ‘normalizing’ and ‘accepting’ the experience of biographical breakdown. A confrontation with other people's story, whether from the live voice of people or mediated by written texts, and with experiences of acceptance at different steps, could indeed greatly help patients to make peace with themselves and with what happened. The use of narratives offers great possibilities, still only partially explored by clinical research.

Finally, the concept of positioning valorizes the relevant and fluid character of identity, permitting participants to let emerge new perspectives for agency and performance of the identity movements.^55^

### 5.1 Clinical implications

This research furnishes a contribution to the literature concerning the study of identity, body and relationships in the field of brain injury and neurological rehabilitation. It provides useful findings for the practitioners who operate in the field of rehabilitation services, as well as for the people and families involved in these kinds of health conditions. A concrete outcome might be represented by implementing the dialogue between disciplines and services and the services of psychological counselling and support for persons with ABI and their families. This might engage the often‐involved families' experiences of abandonment or difficulties over a long period. Also, it could be possible to promote psychological pathways generative of new meanings and potential resources and other more limiting and restrictive aspects of the biography in the specific event of ‘brain damage’, saturating the identity and the possibilities of coping and of construction of new roles and perspectives. In other areas of mental health, people who have had significant problems and who have achieved a high level of adjustment are increasingly valued in the role of expert by experience (i.e., a person who has emotionally processed what has happened to them and is able to share their story with others. Research shows that the presence of experts with experience in mental health services is crucial to the implementation of reasonable hope and cautious optimism, even on the part of those experiencing a similar problem. We believe that these kinds of narrative devices can also be an important resource to positively influence stories of suffering and build confidence, both in patients and family members.^56^

### 5.2 Limitations

Among the limitations of the research is that it did not include the possibility of interviewing more people to facilitate dialogue in the face of specific difficulties. For future developments, mention should be made of research that can take even more specific responsibility for the concrete interaction between disciplines as well as the inclusion of other types of ABI. As a whole, the research considered people of quite different ages who had suffered a stroke 1–5 years earlier. We are aware of the differences in impact related to the stroke event at various stages of life, particularly at a young age.^57^ Future research could differentiate the analysis of placements according to age at the time of stroke and could focus attention on the various stages of the acceptance process and the psychological strategies best suited to facilitate it, including, among others, narrative.

## AUTHOR CONTRIBUTIONS

The first author contributed substantially to the conception and design of the work, while the last author created the structure, collected the data and drafted the paper. They revised it critically for important intellectual content. The third and fourth authors work in the facility at which the research was conducted, codesigned the research, and helped recruit participants. All listed authors have agreed to the final submitted version.

## CONFLICT OF INTEREST STATEMENT

The authors declare no conflict of interest.

## ETHICS STATEMENT

Approval for the study was obtained from the Ethical Committee of the School of Padova, at the University of Padova (Approval No. 3793). All patients provided written informed consent before enrolment in the study. The participants and their families were informed of their right to withdraw at any time. They signed a written informed consent form regarding their participation in the research and its publication.

## Data Availability

The data that support the findings of this study are available on request from the corresponding author. The data are not publicly available due to privacy or ethical restrictions.
